# Radiographic Predictors of Spinal Column Instability After Gunshot Wounds: A Pragmatic Score

**DOI:** 10.7759/cureus.109216

**Published:** 2026-05-19

**Authors:** Nicholas P Derrico, Tyler X Giles, Katherine E Baker, Thomas R Hemphill, Evan C Bowen, John Wilkinson, Gregory R Vance, Madhav Sankhyan, Kenneth A Winter, Olivia Patch, Seth T. Lirette, Chad W Washington, Zachary S Smalley, Jared J Marks

**Affiliations:** 1 Neurosurgery, University of Mississippi Medical Center, Jackson, USA; 2 Radiation Oncology, University of Mississippi Medical Center, Jackson, USA; 3 Radiology, University of Mississippi Medical Center, Jackson, USA

**Keywords:** ballistics, gunshot injuries, neurotrauma, operative management, severity scoring, spine trauma

## Abstract

Background

Firearm injuries to the spinal column remain a major public health concern in the United States and present a challenge in assessing spinal stability. Currently, no scoring system exists to evaluate ballistic injuries to the spinal column. We propose the Ballistic Assessment in Spine Trauma (BLAST) score to differentiate stable from unstable ballistic spine fractures for the purpose of operative stabilization.

Methods

Over approximately 14 years, the neurosurgical service at the University of Mississippi Medical Center evaluated 353 gunshot wounds (GSWs) to the spine. Patients with radiographic injury to the spinal column were included and evaluated using the BLAST score in this retrospective chart review study.

Results

Our results indicate that a score of 6 or greater correlates with operative management. Overall, the BLAST score performed comparably to the accuracy reported in validation studies of the Subaxial Cervical Spine Injury Classification System (SLICS), Thoracolumbar Injury Classification and Severity Scale (TLICS), and Spinal Instability Neoplastic Scale (SINS). The BLAST score achieved receiver operating characteristic (ROC) area under the curve (AUC) values of 0.965 and 0.812 for cervical and thoracolumbar injuries, respectively. The scoring system also demonstrated kappa statistics of 0.39 and 0.40 and intraclass correlation coefficients (ICCs) of 0.82 and 0.79 for the cervical and thoracolumbar spine, respectively.

Conclusion

The BLAST score offers an efficient, accurate, and pragmatic composite scoring system that can differentiate stable from unstable spinal injuries caused by GSWs.

## Introduction

Firearm injuries remain a persistent and critical public health concern in the United States. In 2022, 48,204 individuals died from firearm-related injuries in the United States [1]. An additional 67,000 people are estimated to be injured by firearms each year [2]. The state of Mississippi has specifically seen the highest age-adjusted death rate among all 50 states from 2020 to 2022, with 29.6 deaths per 100,000 population and a total of 848 deaths in 2022 [3]. These injuries also represent a major cost to the US healthcare system. A 2005-2015 retrospective study found that government programs covered 41.6% of the cost burden of firearm-related admissions. Among 317,479 injuries, total costs reached $8.65 billion [4].

While most gunshot wounds (GSWs) present with clearly stable injuries that do not require surgical stabilization, other injuries are less straightforward. Neurologic deficits have been reported in 33%-92.4% of patients with spinal GSWs. Progressive neurological deficit may indicate the need for surgery [5]. As such, scoring systems have been used in other populations to aid in complex surgical decision-making. Established scoring systems include the Subaxial Cervical Spine Injury Classification System (SLICS) [6], Thoracolumbar Injury Classification and Severity Score (TLICS) [7], and the Spinal Instability Neoplastic Score (SINS) [8]. These systems have demonstrated good to excellent performance in predicting the need for surgery in closed spinal trauma and oncologic populations, respectively. Samuel et al. demonstrated that SLICS matched treatment decisions in 93.6% of nonoperative cases and 96.3% of operative cases, with a score of 3 or less indicating nonoperative management and 5 or more indicating operative management [9]. External validation studies of SLICS have demonstrated its utility, although intraobserver and interobserver reliabilities have been questioned [10-15]. For TLICS, Park et al. reported that 94.9% of patients with a score of 4 or less matched the recommendation for conservative treatment in their study [16]. Likewise, 84.2% of patients with TLICS scores of 4 or more underwent surgical treatment [16]. Other studies have similarly reported the effectiveness of TLICS at approximately 90% in predicting operative and nonoperative management [17,18]. SINS has also proven reliable in predicting the need for surgical intervention, with excellent interobserver and intraobserver reliability [19,20], although the indeterminate score group may contain distinct subgroups [21]. However, currently, no scoring system exists for ballistic injuries to the spinal column.

Ballistic injuries represent a unique mechanism of spinal element disruption that must be considered when evaluating spinal stability. Both the TLICS and SLICS systems incorporate neurological status as a scoring component, effectively using neurologic injury as a proxy measure of spinal column instability. In the setting of closed trauma, this assumption is reasonable. However, in ballistic injuries, the relationship between spinal column injury and neurologic injury is not as straightforward. Clinicians experienced with ballistic spinal cord injuries will note the not-uncommon occurrence of spinal cord injury in the absence of any radiographic spinal column injury, presumably due to blast injury to neural tissue as the projectile passes through the body. Our institutional approach to these injuries has therefore been to treat neurologic injury from ballistic trauma and spinal column instability as essentially separate problems.

We propose a pragmatic scoring system, the Ballistic Assessment in Spine Trauma (BLAST) score, to evaluate ballistic spinal column instability requiring operative stabilization. The score is based on anatomic findings from admission computed tomography (CT) imaging obtained during the Advanced Trauma Life Support secondary survey. While SLICS and TLICS rely on magnetic resonance imaging (MRI) to characterize disco-ligamentous injury, MRI is not readily available in patients with retained ballistic fragments and was therefore not incorporated into the score. We also did not include neurologic status in our scoring system, given concern for a variable relationship between neurologic deficit and spinal column stability. Over approximately 14 years, the neurosurgical service at the University of Mississippi Medical Center evaluated 353 GSWs with radiographic injury to the spinal column. Here, we present the scoring system and its validation study.

This paper was previously published as a pre-print on Research Square. 

## Materials and methods

The University of Mississippi Medical Center neurotrauma database was queried for ballistic spinal injuries from 2010 to 2023. This retrospective chart review study was approved by the University of Mississippi Medical Center Institutional Review Board, including a waiver of consent (Approval Number: UMMC-IRB-2023-329). The study was conducted in accordance with the World Medical Association Declaration of Helsinki and all relevant laws and institutional guidelines. Privacy of human subjects was maintained. This study was also conducted in accordance with the EQUATOR Strengthening the Reporting of Observational Studies in Epidemiology (STROBE) guidelines. To be included, patients had to present with radiographic evidence of spinal column injury on admission CT imaging. CT images were reviewed by three neurosurgery residents who had completed junior residency training and their major emergency room call experience. BLAST scores were calculated while blinded to surgical management outcomes, and interrater reliability was assessed using kappa statistics and intraclass correlation coefficients (ICCs). Patients who underwent operative stabilization for spinal column instability, either during the index admission or at follow-up, were considered surgical. Those who did not receive surgical stabilization for spinal injuries were considered non-surgical. Non-surgical patients were typically managed with bracing, per the treating surgeon’s preference. Procedures performed for the removal of metal fragments, management of cerebrospinal fluid fistula, or decompression of neural elements were excluded from the analysis. Therefore, the BLAST score was validated against clinical decision-making by treating surgeons in a real-world setting.

**Figure 1:**
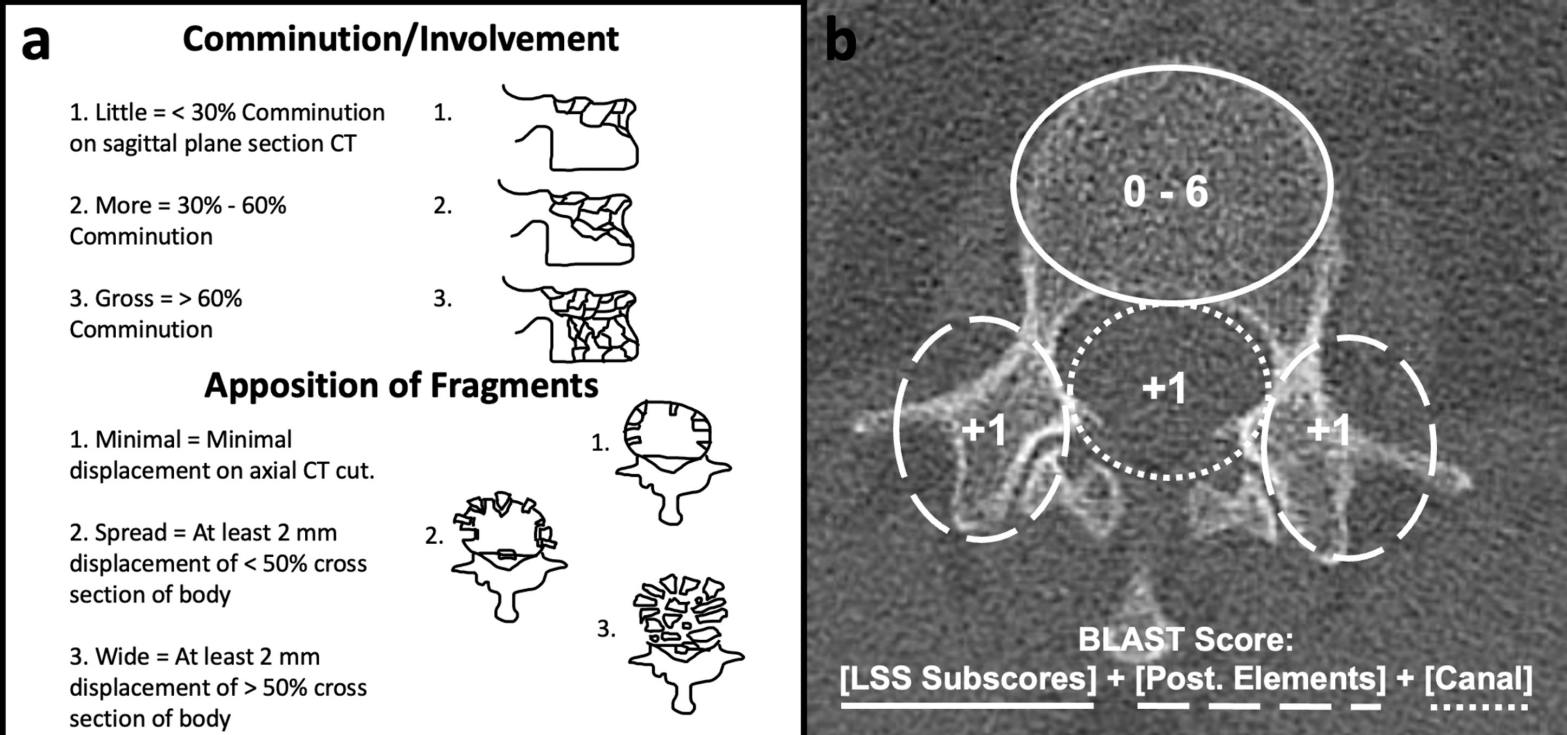
(a) Comminution and apposition for Load-Sharing Score (LSS). (b) Ballistic Assessment in Spine Trauma (BLAST) scoring system. (a) Higher comminution results in a higher injury score, with 3 being the highest. Higher apposition of fragments also results in a higher injury score, with 3 being the highest. (b) Anterior column LSS subscores (encircled by solid line) are scored 0-6 based on comminution (0-3) and apposition (0-3) of fragments. The posterior elements (encircled by a dashed line) and canal (encircled by a dotted line) each add 1 point each when involved. Image Credit: This figure was created using Microsoft PowerPoint (Microsoft Corp., Redmond, WA, USA). No AI was used.

The BLAST score is a simple two-component score applied to subaxial cervical and thoracolumbar spinal injuries. The first component grades the degree of vertebral body injury and is derived from the apposition and comminution subscores of the McCormack Load-Sharing Score (LSS) [22]. Scores for this component range from 0 (no vertebral body injury) to 6 (gross comminution and wide apposition). The sagittal deformity subscore of the LSS was not used due to the lack of upright radiographs in most patients. The second component is a simple tally of involved facet/pedicle complexes and canal involvement, with 1 point assigned for each. Scores range from 0 (no posterior element injury) to 3 (bilateral pedicle or facet complex injury with canal violation). Spinous processes, laminae, and transverse processes were considered non-structural elements and were not included in the score. The total BLAST score therefore ranges from 1 to 9 in included patients (Figure 1).

Receiver operating characteristic (ROC) curves with 95% confidence intervals (CIs) were generated, and the area under the curve (AUC) was calculated. This was used to identify a cutoff score of 6, at which likelihood ratios, sensitivity, and specificity were maximized. At this threshold, the BLAST score demonstrated its best performance in predicting operative and nonoperative management. Based on this cutoff, 95% CIs were calculated. The BLAST score’s prediction of operative management was used as a proxy for spinal instability, given that this is the predominant indication for operative intervention, although this is an imperfect surrogate. Analysis was performed using GraphPad Prism (Dotmatics, Boston, MA, USA), and figures and graphs were produced using both GraphPad Prism and PowerPoint (Microsoft Corp., Redmond, WA, USA).

## Results

Our cohort included 353 patients with ballistic spinal column injuries from 2010 to 2023, of whom 62 cases involved the subaxial cervical spine and 294 involved the thoracolumbar spine. Three patients had injuries involving both the cervical and thoracolumbar spine; these were counted as two separate injuries. A total of 259 (73.4%) patients had at least one follow-up visit. The average age was 28.9 years (range 2-69), and 243 (68.8%) were male. Fifty-five (15.6%) patients were children under the age of 18.

**Figure 2:**
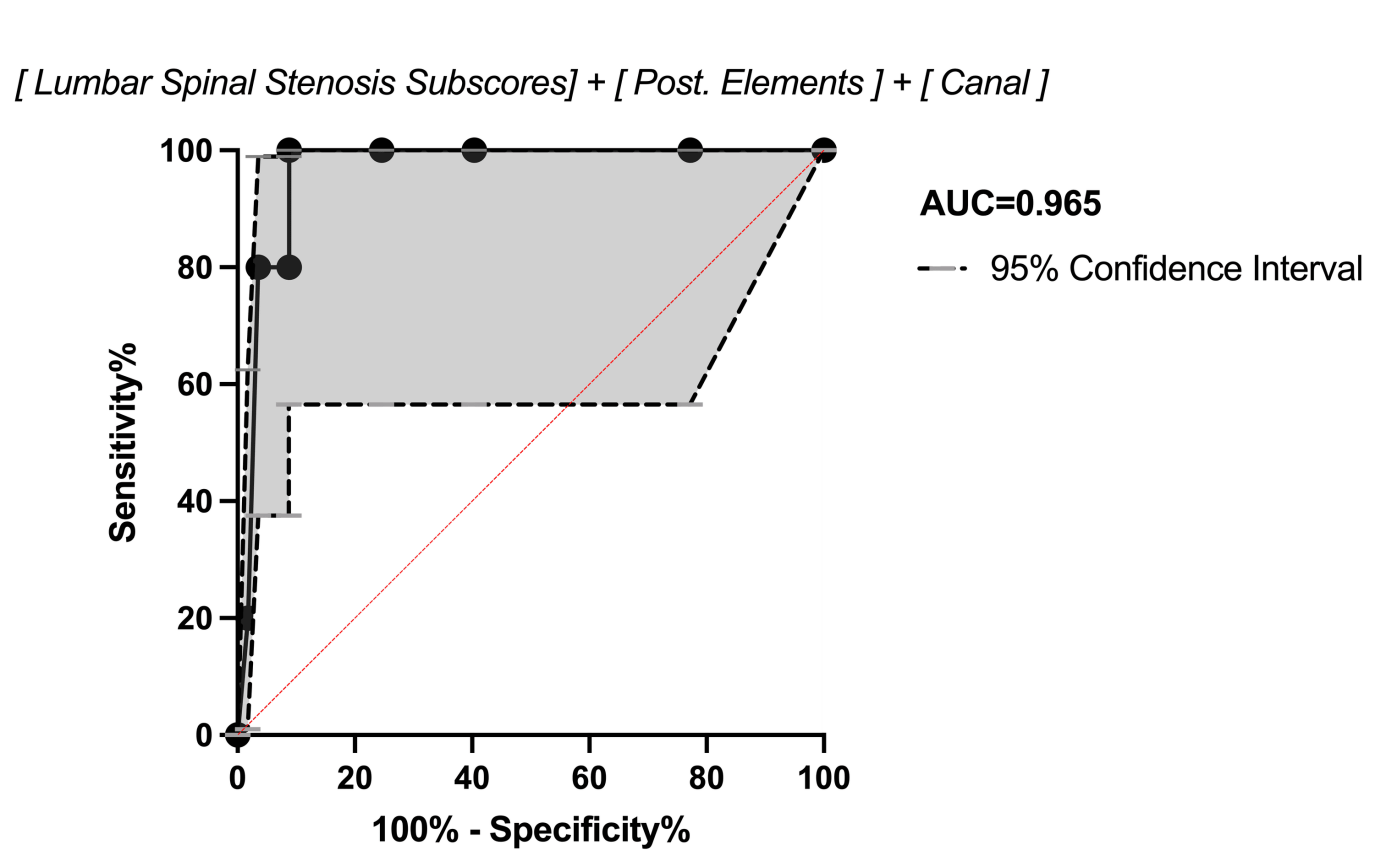
ROC curve for subaxial cervical composite score. AUC: area under the curve, ROC: receiver-operating characteristic.

Of the 62 cases involving the subaxial cervical spine, 57 were managed nonoperatively, and 5 underwent surgical stabilization (Table 1). Among the 57 nonoperative cases, the average apposition subscore was 0.30, the average comminution subscore was 0.21, and the average posterior element subscore was 1.14. The average total BLAST score in this group was 1.65. Among the five operative cases, the average apposition subscore was 2.00, the average comminution subscore was 2.00, and the average posterior element subscore was 1.80. The average total BLAST score in operative cases was 5.80. The ROC curve demonstrated an AUC of 0.965 (Figure 2), and a positive likelihood ratio of 22.8 when a score of 6 or greater indicated surgical management.

**Table 1:** Average comminution and apposition scores, vertebral body and posterior element involvement, and average total BLAST scores with standard deviations. BLAST: Ballistic Assessment in Spine Trauma.

	Total (n)	Vertebral body involvement	Average apposition score	Average comminution score	Posterior elements involvement	Average posterior elements score	Average total BLAST score
Cervical nonoperative	57	9 (15.8%)	0.30	0.21	41 (71.9%)	1.14	1.65
Standard deviation	-	-	0.71	0.49	-	1.18	1.58
Cervical operative	5	5 (100%)	2.00	2.00	5 (100%)	1.80	5.80
Standard deviation	-	-	0.00	0.63	-	0.63	0.98
Thoracolumbar nonoperative	283	100 (35.3%)	0.59	0.51	219 (77.4%)	1.39	2.49
Standard deviation	-	-	0.69	0.58	-	1.24	1.52
Thoracolumbar operative	11	8 (72.7%)	1.27	1.18	10 (91.0%)	2.27	4.73
Standard deviation	-	-	0.86	0.72	-	1.44	1.86

**Figure 3:**
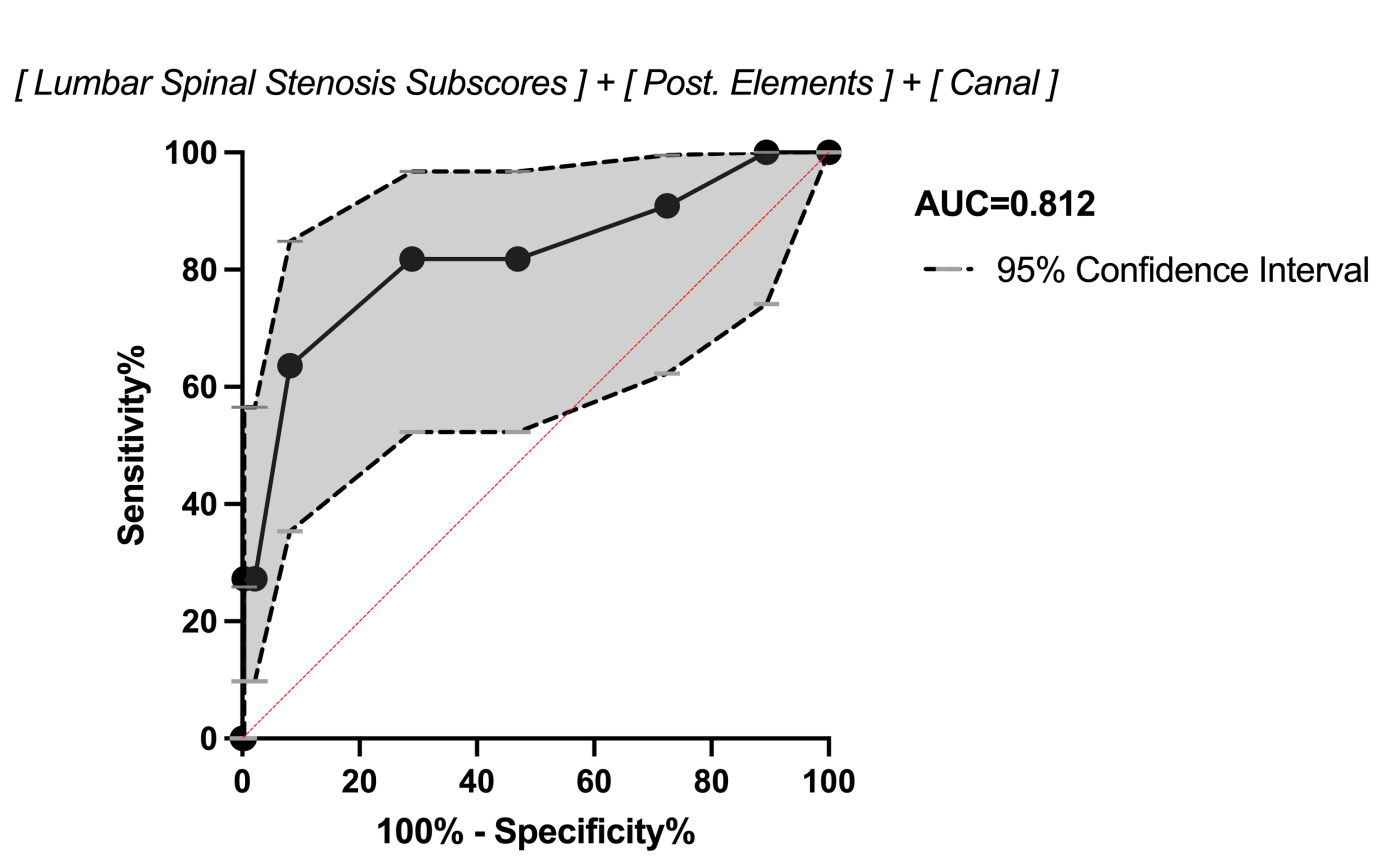
ROC curve for thoracolumbar composite score. AUC: area under the curve, ROC: receiver-operating characteristic.

A total of 294 cases involved thoracolumbar injuries. Of these, 283 were managed nonoperatively, while the remaining 11 underwent surgical treatment. For nonoperative cases, the mean apposition subscore was 0.59, the mean comminution subscore was 0.51, and the mean posterior element subscore was 1.39 (Table 1). Operative thoracolumbar cases had a mean apposition subscore of 1.27, a mean comminution subscore of 1.18, and a mean posterior element subscore of 2.27. On average, nonoperative thoracolumbar patients had a total score of 2.49, while operative thoracolumbar patients had a total score of 4.73. The ROC curve demonstrated an AUC of 0.812 for thoracolumbar ballistic injuries (Figure 3). Finally, the BLAST score yielded a positive likelihood ratio of 12.86 when a score of 6 or greater indicated surgical treatment, and 77.18 when a score of 7 or greater indicated surgical treatment.

The BLAST scores were calculated by three neurosurgery residents experienced in the clinical evaluation of these patients and the interpretation of their imaging. Interrater reliability within the BLAST score was determined via a kappa statistic and ICC. For the cervical spine, the kappa statistic was 0.39, and the ICC was 0.82. For the thoracolumbar spine, the kappa statistic was 0.40, and the ICC was 0.79. 

## Discussion

The BLAST score represents an initial step toward developing a scoring system to assess spinal column instability in ballistic fractures of the subaxial cervical and thoracolumbar spine. A score of 6 or greater may appropriately suggest the need for surgical treatment in both cervical and thoracolumbar injuries. The subaxial cervical cohort, in particular, is statistically fragile due to the small number of operative cases included; therefore, these findings should be interpreted with caution. Based on ROC AUC values, the BLAST score discriminates between stable and unstable injuries comparably to the well-established SLICS, TLICS, and SINS scoring systems. Thus, the BLAST score represents a viable adjunct in the evaluation of ballistic spinal column injuries, to be used alongside clinical judgment and multidisciplinary review.

The BLAST score is also simple enough to generate reproducible results between observers. For the cervical spine, the kappa statistic was 0.39, and the ICC was 0.82; for the thoracolumbar spine, the kappa statistic was 0.40, and the ICC was 0.79. While some variability may exist in assessing the comminution and apposition subscores, we observed fair kappa values and good ICC values, which are comparable to those reported for other scoring systems. A 2020 review of SLICS found interrater reliability kappa values between 0.15 and 0.20 and ICC values between 0.41 and 0.79 [15]. TLICS has reported kappa values of 0.69 [22] and 0.868 [17], with no ICC reported to our knowledge. SINS has reported interrater reliability of κ = 0.76 [19] and 0.846 [20]. However, a separate 2019 review of SINS reported interrater reliability kappa values between 0.38 and 0.53 and ICC values between 0.55 and 0.99 [23].

Our study has several limitations. The score was derived from a single institutional cohort, and a true validation cohort would require an independent patient population. Given the relatively low rate of surgical management, we were concerned that splitting the cohort into derivation and validation groups would reduce the statistical power of the analysis. External validation studies are therefore needed to further evaluate the BLAST score. The BLAST score does not account for neurological status. While neurologic status is an important consideration in patient management, its relationship to spinal column stability is not clearly defined and was excluded by design. Our study is also limited by a substantial proportion of patients lost to follow-up. Although this may introduce bias, a follow-up rate of 73.4% exceeds that reported in other studies, where loss to follow-up ranges from 41.4% to 69% among gun violence survivors [24,25]. Our institutional protocol for early assessment of bracing failure includes radiographic surveillance at six weeks post-injury, which 64.7% of patients completed. However, longer-term outcomes related to motor recovery and functional status were not consistently available. Additionally, exclusion of MRI due to retained ballistic fragments may limit assessment of disco-ligamentous injury. Finally, firearm type was not consistently available. We suspect that most injuries were caused by handguns, consistent with civilian level I trauma center patterns in which handgun-related injuries predominate [26]. However, higher-energy weapons such as rifles may produce different injury patterns, limiting generalizability. Further validation in external cohorts will be necessary to strengthen and expand the applicability of the BLAST score.

## Conclusions

The BLAST score offers an efficient and pragmatic tool that can differentiate operative from nonoperative fractures in the unique setting of GSWs to the spine. Our results indicate that a score of 6 or greater predicts operative management in a real-world cohort. The BLAST score demonstrates similar effectiveness and reproducibility to the SLICS, TLICS, and SINS scoring systems in predicting the need for operative stabilization of fractures. This score may be a useful tool for evaluating these injuries in centers where they are encountered infrequently. We hope that additional research in other cohorts will further validate this scoring system.
